# Development of a microarray for two rice subspecies: characterization and validation of gene expression in rice tissues

**DOI:** 10.1186/1756-0500-7-15

**Published:** 2014-01-08

**Authors:** Jia-Shing Chen, Shang-Chi Lin, Chia-Ying Chen, Yen-Ting Hsieh, Ping-Hui Pai, Long-Kung Chen, Shengwan Lee

**Affiliations:** 1 Phalanx Biotech Group, 6F, No.6, Technology Road 5, Hsinchu Science Park, Hsinchu 30078, Taiwan, Republic of China

**Keywords:** *Japonica*, *Indica*, Rice, Microarray

## Abstract

**Background:**

Rice is one of the major crop species in the world helping to sustain approximately half of the global population’s diet especially in Asia. However, due to the impact of extreme climate change and global warming, rice crop production and yields may be adversely affected resulting in a world food crisis. Researchers have been keen to understand the effects of drought, temperature and other environmental stress factors on rice plant growth and development. Gene expression microarray technology represents a key strategy for the identification of genes and their associated expression patterns in response to stress. Here, we report on the development of the rice OneArray® microarray platform which is suitable for two major rice subspecies, *japonica and indica*.

**Results:**

The rice OneArray® 60-mer, oligonucleotide microarray consists of a total of 21,179 probes covering 20,806 genes of *japonica* and 13,683 genes of *indica*. Through a validation study, total RNA isolated from rice shoots and roots were used for comparison of gene expression profiles via microarray examination. The results were submitted to NCBI’s Gene Expression Omnibus (GEO). Data can be found under the GEO accession number GSE50844 (http://www.ncbi.nlm.nih.gov/geo/query/acc.cgi?acc=GSE50844). A list of significantly differentially expressed genes was generated; 438 shoot-specific genes were identified among 3,138 up-regulated genes, and 463 root-specific genes were found among 3,845 down-regulated genes. GO enrichment analysis demonstrates these results are in agreement with the known physiological processes of the different organs/tissues. Furthermore, qRT-PCR validation was performed on 66 genes, and found to significantly correlate with the microarray results (R = 0.95, p < 0.001***).

**Conclusion:**

The rice OneArray® 22 K microarray, the first rice microarray, covering both *japonica* and *indica* subspecies was designed and validated in a comprehensive study of gene expression in rice tissues. The rice OneArray® microarray platform revealed high specificity and sensitivity. Additional information for the rice OneArray® microarray can be found at http://www.phalanx.com.tw/index.php.

## Background

Rice is one of the most important crops in the world - a staple food supporting more than half of the world’s 7 billion people. By 2050, the global population is anticipated to expand between 7.5 and 10.5 billion with the growth concentrated mainly in rice consuming countries. According to a 2009 report by the United Nations Food and Agriculture Organization (FAO), the world will have to produce 70% more food by 2050 to feed a projected extra 2.3 billion people. As such, rice crop production will play an important role to maintain food security in the coming future.

In recent years due to abnormal climate changes, several grain countries such as Australia, Brazil, and Thailand, have suffered frequently from devastating floods and droughts, resulting in global grain crop losses and food price inflation. Additionally, extreme weather events have occurred with more frequency throughout the Asia Pacific region; for example, Typhoon Morakot brought catastrophic damage to Taiwan in 2009 [1].

Furthermore, substantial decreases in rice yields due to increased nighttime temperatures associated with global warming [2], and increased minimum air temperatures during growing seasons have been reported in China and Philippines with predictions of this phenomenon continuing [2,3]. Increasing in atmospheric brown clouds and greenhouse gas has been proposed to reduce historical rice harvests in India well below expected levels. These studies have profound implications for ongoing and future efforts for climate and air quality improvements [4,5].

Therefore, the development of new rice strains against the threat of climate change and water shortages is an important issue of food security for the coming future [6,7]. Traditional rice breeding techniques required upwards of 10 years to develop new rice strains due to a multi-generational process of selecting and preserving strain variety in the offspring. The application of molecular marker-assisted breeding will facilitate the pyramiding of desirable alleles at multiple loci and shorten the time needed for developing new varieties [8]. An increase in the fundamental knowledge of rice biology (e.g. seed formation, disease resistance, growth etc.) is necessary to provide for effective strategies to improve crop yield and production. Nevertheless, the majority of the rice consumption is produced by *indica* subspecies, but the greater part of the genomic work is done on *japonica*. To overcome the imbalance in rice related studies, it is important to generate a microarray using in both subspecies.

To aid rice researchers and plant biologists, the rice OneArray® gene expression microarray was developed by Phalanx Biotech Group Inc. Based on the rice genome sequences from the Rice Genome Annotation Project (version 6.1) and the Beijing Genome Institute 2008 database, probes were designed and selected to cover 90% of the well-annotated Gene found on both *japonica* and *indica* subspecies. Researchers will find the rice OneArray® microarray suitable for large-scale basic studies, stress physiology research, and biomarker discovery.

In this paper, we report the development and validation of the 22 k rice OneArray® oligo-microarray platform manufactured by Phalanx Biotech Group, Inc. The high data reproducibility on technical replicates and the platform’s capacity to identify differential gene expression in different tissues and rice subspecies was demonstrated.

## Results and discussion

### Rice OneArray® gene selection and probe design

**Figure 1:**
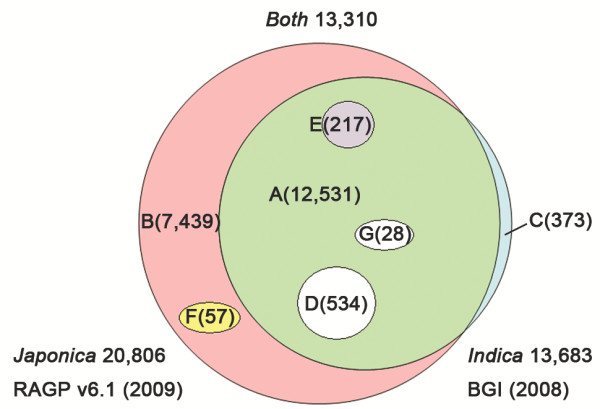
**Number of probes in the microarray represented in *japonica *and *indica *subspecies.** A total of 21,179 probes were classified into seven groups **(A, B, C, D, E, F and G)** based on Blast analysis against corresponding genomes of both subspecies. For japonica, 20,806 probes were represented among **A**, **B**, **D**, **E**, **F**, and **G** groups, with single hit probes in group **A**, **B**, and **D**, and multiple-hits probes in group **E**, **F**, and **G**. For indica, 13,683 probes were incorporated in **A**, **C**, **E**, **D**, and **G** groups, with single hit probes in group **A**, **C**, and **E** and multiple hits probes in group **D** and **G** group.

The primary goal of this project was to develop a rice microarray platform to study gene expression patterns relevant to important biological and physical controls across *japonica* and *indica* subspecies. The rice OneArray® microarray was specifically designed to cover important regulatory pathways and to include genes involved in the biological function of chloroplasts, oxidative stress, grain quality, nitrogen phosphate, sugar synthesis, photosynthesis, plant hormone, anther development, and transcription factors. 9719 *japonica* target genes were curated and selected from a compilation of databases including Michigan State University database (ftp://ftp.plantbiology.msu.edu/pub/data/Eukaryotic_Projects/o_sativa/annotation_dbs/pseudomolecules/chloroplast.dir/chrC.cDNA), GOSlim (http://rice.plantbiology.msu.edu/annotation_pseudo_goslim.shtml), K E G G (http://www.kegg.jp/kegg-bin/show_organism?menu_type=pathway_maps&org=osa), Gramene (ftp://ftp.gramene.org/pub/gramene/pathways/ricecyc/), and PlnTFDB (http://plntfdb.bio.uni-potsdam.de/v3.0/). mRNA transcript sequences of each *japonica* target gene were first subjected for microarray probe design by using IMPORT software (Industrial Technology Research Institute of Taiwan, R.O.C). Probes were designed according to the following criteria; 60 nucleotides in length, GC% between 40-60%, fewer than 6 simple nucleotide repeats, and probe location within 1200 bp from 3’ terminus. To remove the probes with non-specific binding and strong secondary-structure, probe sequences were run through Blast analyses against *japonica* (from Rice Genome Annotation Project (version 6.1)) and *indica* whole genome sequences (from BGI; Beijing Genome Institute). Based on the above probe design procedure, 92% of 9719 *japonica* target genes and 86% of 8995 *indica* target genes were selected for inclusion on the rice OneArray® microarray. The probe set was further supplemented with probes against well-annotated genes as defined by Gene Ontology. In total, 21,179 probes (Figure 1) were selected and designed based on the rice whole genome sequences from *japonica* and *indica* subspecies, plus 824 control probes including IHC (Intrinsic Hybridization Control), IHL (Intrinsic Hybridization Ladder), ITQC (Intrinsic Target Quality control), and negative controls. In summary, the rice OneArray® microarray is suitable for the detection of 20,806 genes of *japonica* and 13,683 genes of *indica*.

### Array quality

**Figure 2:**
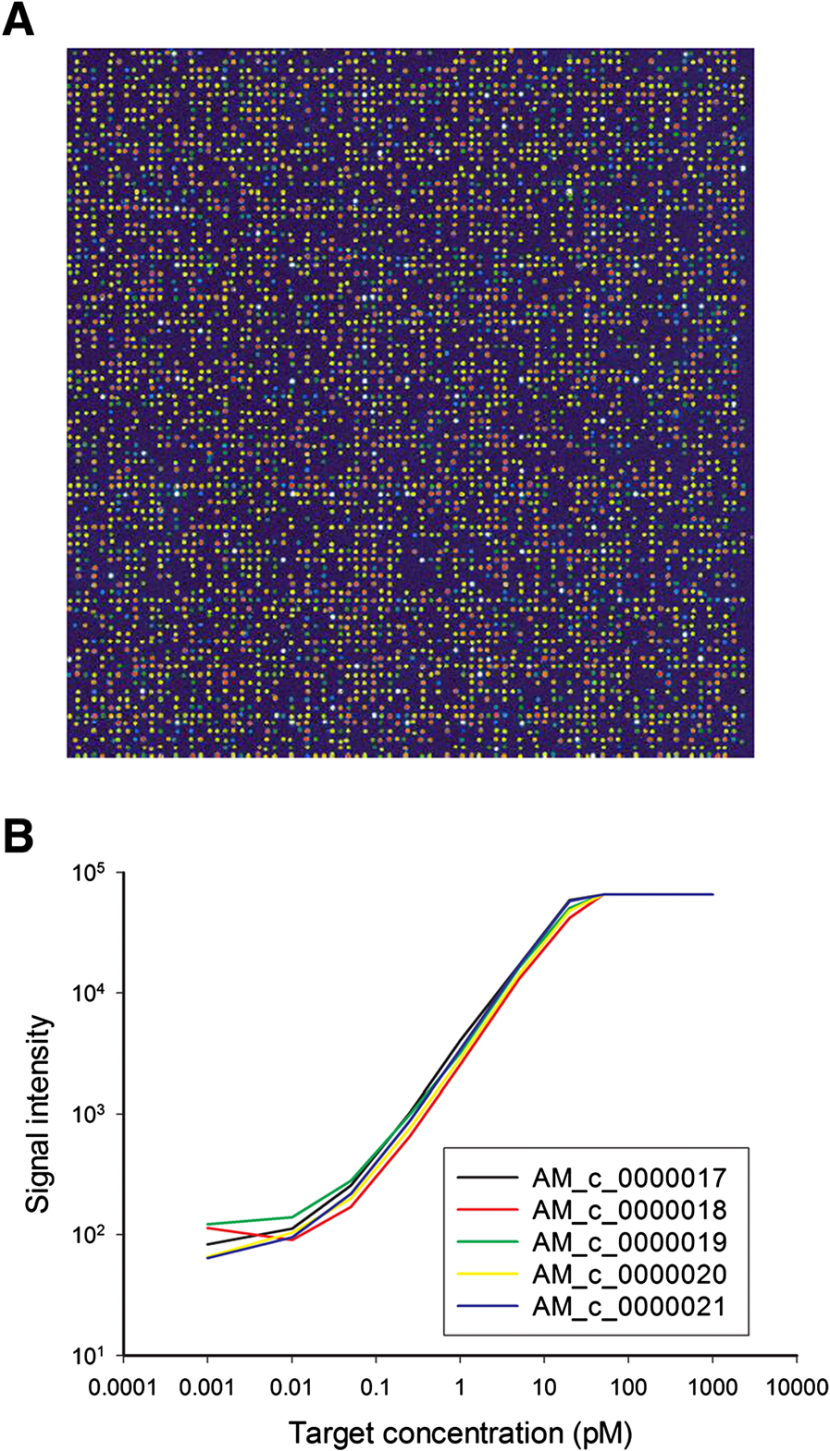
**Validation of Rice OneArray ® microarray quality. A**. Examination of spot quality control. **B**. Sensitivity testing by using five external spike-in probes.

Each microarray undergoes a spot QC process to evaluate probe deposition and immobilization efficiency. In brief, microarrays were incubated with random, 10-mer oligo probes labeled with Cy3 using the standard hybridization protocol (Figure 2A). The sensitivity and dynamic detection range of the rice OneArray miroarray were tested using commercially available external control probe sets (external spike-in system) supplemented with 10 μg Cy5-labeled aRNA of rice shoot. The results showed that the minimal detectable concentration of probes was approximately 0.05 pM (Figure 2B). In summary, the rice microarray demonstrated high sensitivity and dynamic range in this gene expression profiling study.

### Rice OneArray® microarray technical performance

The Phalanx microarray platform is based upon the hybridization of a single labeled sample (derived from RNA), followed by one-channel detection. The intensity of the hybridization signal is used to determine target concentration. In order to validate the technical quality of each probe in our arrays, we carried out 10 independent hybridizations on samples representing two different rice tissues – root and shoot.

**Figure 3:**
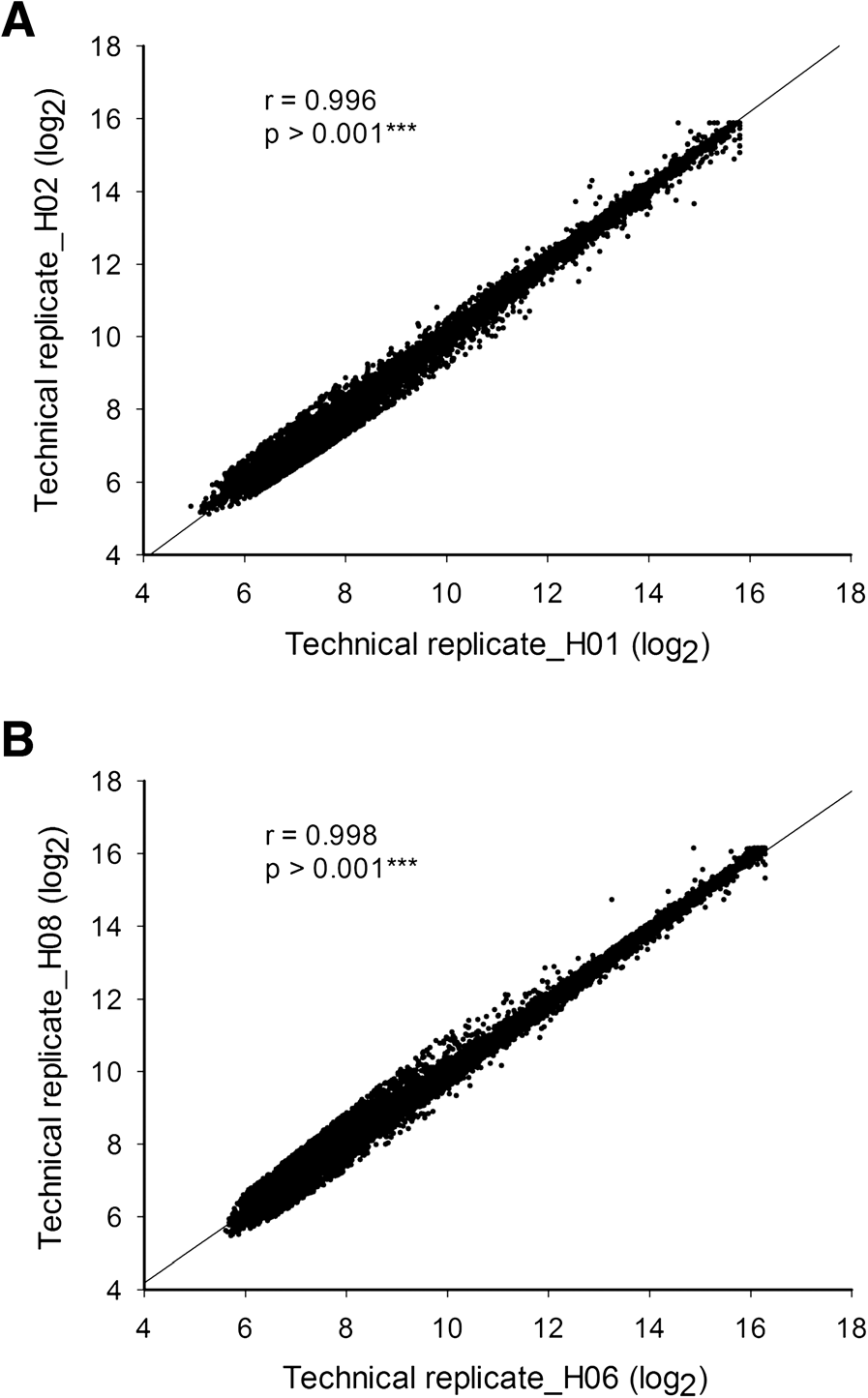
**Rice OneArray® microarray reproducibility.** Scatter plot indicating correlation between microarray technical replicates. High correlations were found between all technical replicates. **A**. rice shoot (r = 0.996, p < 0.001***). **B**. rice root (r = 0.998, p < 0.001***).

To examine the gene expression profiles between rice root and shoot development, total RNA extracted from rice root and shoot were processed on the rice OneArray® microarray following the standard protocol using five arrays for each tissue type. Raw expression data from 10 microarrays (e.g. 5 arrays × 2 tissues) were normalized and Pearson’s correlation coefficients were calculated for the data sets of hybridization signal intensities. All normalized and raw data were submitted to NCBI’s Gene Expression Omnibus (GEO) for others to examine. The data are accessible via GEO Series accession number GSE50844 http://www.ncbi.nlm.nih.gov/geo/query/acc.cgi?acc=GSE50844. It was demonstrated that the average spot number of each tissue was approximately 18,000 spots with an average signal intensity of each spot of 3,000. High correlation coefficients were obtained in all cases and the results obtained for the rice root and shoot sample are shown in Figure 3A (r = 0.996; p-value <0.001***) and 3B (r = 0.998; p-value <0.001***) respectively. Furthermore, the significant correlation was observed between technical repeats (Table 1, R > 0.983) in each of the 5 arrays. In summary, 90% of probes can be detected in rice root and shoot tissue, the detectable spot percentage is higher than other species array data (60-70%). The higher coverage rate of the rice OneArray® microarray may be attributed to the comprehensive target gene selection of genes relevant to rice development. These results also demonstrate high correlation between different technical experiments underlining the high precision manufacturing of the rice OneArray® microarray platform.

**Table 1:** The correlation between technique repeats of rice shoot and root

				**Shoot**					**Root**		
		**H01**	**H02**	**H03**	**H04**	**H05**	**H06**	**H07**	**H08**	**H09**	**H10**
	**H01**	**1.000**	**0.989**	**0.987**	**0.986**	**0.985**	0.670	0.673	0.674	0.675	0.672
	**H02**		**1.000**	**0.991**	**0.985**	**0.988**	0.678	0.678	0.681	0.682	0.678
**Shoot**	**H03**			**1.000**	**0.987**	**0.988**	0.681	0.679	0.683	0.681	0.678
	**H04**				**1.000**	**0.983**	0.667	0.673	0.671	0.675	0.671
	**H05**					**1.000**	0.674	0.679	0.677	0.681	0.677
	**H06**						**1.000**	**0.984**	**0.994**	**0.987**	**0.983**
	**H07**							**1.000**	**0.987**	**0.993**	**0.985**
**Root**	**H08**								**1.000**	**0.990**	**0.985**
	**H09**									**1.000**	**0.986**
	**H10**										**1.000**

### Comparison of gene expression in rice root and shoot using gene ontology analysis

To elucidate the genes regulating rice tissue development, comparisons between shoot and root gene expression profiles from the rice OneArray® microarray were normalized and analyzed using Rosetta software. A list of differentially expressed genes was generated; among 3,138 up-regulated genes (Additional file 1), 438 were shoot-specific genes (Additional file 2), and among 3,845 down-regulated genes (Additional file 3), 463 were root-specific genes (Additional file 4). Gene set analysis was performed using Gene Ontology terms with functional annotation, as described in DAVID Bioinformatics Resources 6.7 (http://david.abcc.ncifcrf.gov/) [9,10]. First, this method detects significantly up- or down-regulated clusters of functionally related genes in lists ordered by differential expression. Annotated genes in these different groups were then classified into different GO biological processes and the percentages of differential gene expressions were calculated for each process. Among up-regulated genes, GO biological processes included oxidation reduction (4.33%), photosynthesis (1.15%), pigment metabolic (0.41%) and biosynthetic process (0.38%), and fatty acid metabolic (0.48%) and biosynthetic processes (0.45%). A similar set of GO processes were observed in the shoot-specific cluster (Table 2). Among down-regulated genes, GO biological processes included regulation of transcription (2.86%), response to oxidative stress (0.7%), and cell wall polysaccharide metabolic processes (0.13%), of which the majority was observed in root-specific cluster (Table 3). Overall, these results are in general agreement with the known physiological processes of the different organs/tissues suggesting the rice OneArray® platform is capable of providing reliable gene-expression data.

**Table 2:** Go term significantly represented in up-regulated and shoot-specific gene clusters

	**Term (biological process)**	**Count**	**Percentage**	**P-value**
Up-regulated genes: 3138 (shoot vs. root)	Oxidation reduction	136	4.33%	1.10E-17
	Photosynthesis	36	1.15%	1.56E-15
	Photosynthesis, light harvesting	10	0.32%	2.70E-07
	Pigment metabolic process	13	0.41%	6.27E-06
	Pigment biosynthetic process	12	0.38%	9.67E-06
	Photosynthesis, light reaction	12	0.38%	1.30E-05
	Generation of precursor metabolites and energy	36	1.15%	5.09E-05
	Fatty acid biosynthetic process	14	0.45%	0.00286
	Fatty acid metabolic process	15	0.48%	0.004194
	Electron transport chain	14	0.45%	0.006286
	Aminoglycan metabolic process	7	0.22%	0.006382
	Lipid biosynthetic process	21	0.67%	0.007102
	Cellular aldehyde metabolic process	4	0.13%	0.007622
	Lipid transport	12	0.38%	0.009804
	Lipid localization	12	0.38%	0.009804
Shoot-specific genes: 438	Oxidation reduction	40	9.13%	3.40E-07
	Fatty acid metabolic process	8	1.83%	1.30E-03
	Recognition of pollen	6	1.37%	2.70E-03
	Pollination	6	1.37%	2.70E-03
	Cell recognition	6	1.37%	2.70E-03
	Pollen-pistil interaction	6	1.37%	2.70E-03
	Fatty acid biosynthetic process	7	1.60%	2.80E-03
	Reproductive cellular process	6	1.37%	2.90E-03
	Regulation of transcription	30	6.85%	1.10E-02

**Table 3:** Go term significantly represented in down-regulated and root-specific gene clusters

	**Term (biological process)**	**Count**	**Percentage**	**P-value**
Down-regulated genes: 3845 (shoot vs. root)	Regulation of transcription	110	2.86%	6.28E-13
	Regulation of transcription, NA-dependent	71	1.85%	1.20E-10
	Regulation of RNA metabolic process	71	1.85%	1.48E-10
	Transcription	65	1.69%	8.87E-10
	Response to oxidative stress	27	0.70%	1.93E-08
	Oxidation reduction	81	2.11%	1.21E-05
	Cell wall polysaccharide metabolic process	5	0.13%	0.008210995
	Oxylipin metabolic process	5	0.13%	0.008210995
	Xylan metabolic process	5	0.13%	0.008210995
	Xylan catabolic process	5	0.13%	0.008210995
	Oxylipin biosynthetic process	5	0.13%	0.008210995
Shoot-specific genes: 438	Response to oxidative stress	11	2.51%	4.30E-05
	Oxidation reduction	27	6.16%	1.30E-03
	Cell wall polysaccharide metabolic process	4	0.91%	1.50E-03
	Xylan metabolic process	4	0.91%	1.50E-03
	Hemicellulose metabolic process	4	0.91%	1.50E-03
	Xylan catabolic process	4	0.91%	1.50E-03
	Regulation of transcription	27	6.16%	5.30E-03
	Polysaccharide metabolic process	8	1.83%	6.90E-03

### qRT-PCR validation

**Figure 4:**
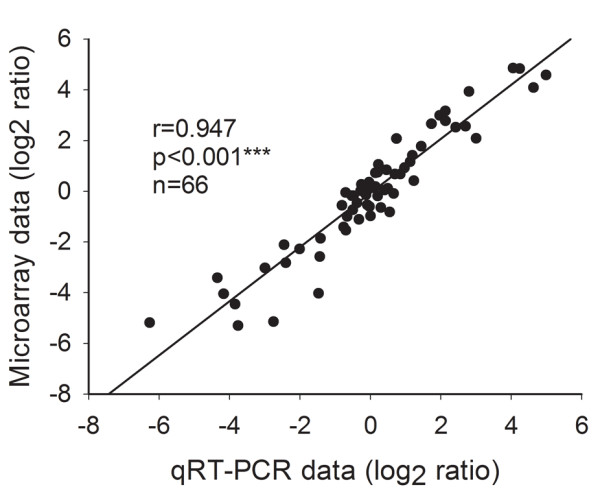
**Correlation between qRT-PCR and Rice OneArray® microarray results.** Statistically significant correlation (r = 0.947, p < 0.001***) was obtained for all 66 tested genes.

To further validate the microarray results, quantitative real time PCR (qRT-PCR) assays were performed on the same RNA samples used for microarray analysis. A total of 66 genes at varying expression levels including up-regulated, down-regulated, and not differentially-expressed were selected for validation by comparison of root and shoot expression profiles. Comparisons between microarray and qRT-PCR data are shown (Table 4), and microarray results correlated well with qRT-PCR validation (Figure 4, R = 0.95, p < 0.001***).

**Table 4:** Comparison of expression levels (log2 ration) from qRT-PCR and microarray for selected target genes

**Target gene**	**MSU Gene ID**	**Microarray value**	**qRT-PCR value**
		**(log2ratio)**	**(log2ratio)**
Zinc finger, C3HC4 type, domain containing protein, expressed	Os07g29600	0.73	0.14
bZIP transcription factor domain containing protein, expressed	Os07g48180	−0.04	−0.7
Ankyrin repeat-rich protein, putative, expressed	Os08g15840	−0.99	−0.66
bZIP transcription factor domain containing protein, expressed	Os08g26880	1.42	1.19
Zinc finger family protein, putative, expressed	Os08g03310	4.58	4.99
MYB family transcription factor, putative, expressed	Os08g33660	3.93	2.8
B-box zinc finger family protein, putative, expressed	Os09g35880	0.93	0.97
Zinc finger, C3HC4 type domain containing protein, expressed	Os09g33670	−0.13	−0.12
MYB family transcription factor, putative, expressed	Os09g36250	0.75	0.2
OsWRKY80 - Superfamily of TFs having WRKY and zinc finger domains, expressed	Os09g30400	−0.17	−0.48
BHLH transcription factor, putative, expressed	Os09g32510	2.66	1.73
Zinc finger, C3HC4 type domain containing protein, expressed	Os09g26400	−0.17	−0.53
Dof zinc finger domain containing protein, putative, expressed	Os09g29960	2.57	2.71
Auxin response factor 18, putative, expressed	Os10g33940	−1.11	−0.32
Transcription factor, putative, expressed	Os12g13170	−0.08	0.66
ZOS12-05 - C2H2 zinc finger protein, expressed	Os12g31840	−0.45	−0.38
OsRR4 type-A response regulator, expressed	Os01g72330	2.53	2.42
Inducer of CBF expression 2, putative, expressed	Os01g70310	4.86	4.05
PHD finger protein, putative, expressed	Os01g66420	0.02	−0.28
SNF2 family N-terminal domain containing protein, expressed	Os02g02290	0.14	0.11
MYB family transcription factor, putative, expressed	Os01g59660	−1.41	−0.76
No apical meristem protein, putative, expressed	Os01g48130	0.31	−0.02
GATA transcription factor 25, putative, expressed	Os02g05510	−0.53	−0.09
PHD finger protein, putative, expressed	Os02g35600	2.08	0.74
AP2 domain containing protein, expressed	Os02g29550	0.42	1.23
Two-component response regulator, putative, expressed	Os02g55320	0.84	0.46
OsMADS22 - MADS-box family gene with MIKCc type-box, expressed	Os02g52340	2.78	2.13
Histidine kinase, putative, expressed	Os02g50480	0.12	0.49
DIRP family protein, putative, expressed	Os03g43800	−0.18	0.21
OsWRKY55 - Superfamily of TFs having WRKY and zinc finger domains, expressed	Os03g20550	−2.11	−2.45
ZOS3-23 - C2H2 zinc finger protein, expressed	Os03g61640	−0.64	0.3
NAC domain-containing protein 67, putative, expressed	Os03g60080	−4.04	−4.17
MYB family transcription factor, putative, expressed	Os04g49450	−2.27	−2.01
Auxin response factor, putative, expressed	Os04g57610	1.17	1.13
MYB_Al protein, putative, expressed	Os04g58020	0.1	−0.12
Zinc RING finger protein, putative, expressed	Os06g03580	−2.82	−2.41
bHelix-loop-helix transcription factor, putative, expressed	Os05g46370	−0.55	−0.81
Transcription factor, putative, expressed	Os05g37170	−2.58	−1.44
myb-like DNA-binding domain containing protein, expressed	Os06g24070	4.09	4.63
Zinc finger protein, putative, expressed	Os06g33810	0.27	−0.25
N-terminal asparagine amidohydrolase, putative, expressed	Os06g41390	−0.6	−0.02
TIP41, putative, expressed	Os03g55270	−0.73	−0.5
B3 DNA binding domain containing protein, expressed	Os01g04800	−3.02	−2.99
ZOS1-03 - C2H2 zinc finger protein, expressed	Os01g04120	−3.41	−4.35
TCP family transcription factor, putative, expressed	Os01g11550	2.09	3
RNA-binding zinc finger protein, putative, expressed	Os07g48410	0.68	0.85
GRAS family transcription factor domain containing protein, expressed	Os07g38030	1.77	1.44
Response regulator receiver domain containing protein, expressed	Os07g49460	−1.54	−0.69
Auxin response factor, putative, expressed	Os08g40900	−0.82	0.55
Homeobox associated leucine zipper, putative, expressed	Os09g21180	1.06	0.23
No apical meristem protein, putative, expressed	Os10g42130	−0.03	−0.19
MYB family transcription factor, putative, expressed	Os01g74410	−4.45	−3.84
bZIP transcription factor domain containing protein, expressed	Os01g55150	0.18	0.15
OsWRKY71 - Superfamily of TFs having WRKY and zinc finger domains, expressed	Os02g08440	−5.14	−2.75
Ethylene-responsive transcription factor, putative, expressed	Os02g43790	−4.02	−1.47
TCP family transcription factor, putative, expressed	Os02g42380	4.84	4.24
RING-H2 finger protein, putative, expressed	Os02g54830	0.35	−0.04
Dof zinc finger domain containing protein, putative, expressed	Os02g47810	2.99	1.97
Ethylene-responsive transcription factor, putative, expressed	Os03g09170	−5.3	−3.76
Homeobox associated leucine zipper, putative, expressed	Os03g08960	3.17	2.13
TCP-domain protein, putative, expressed	Os02g51280	0.05	0.4
Response regulator receiver domain containing protein, expressed	Os03g17570	−1.86	−1.42
ZOS3-21 - C2H2 zinc finger protein, expressed	Os03g60560	−5.18	−6.27
MYB family transcription factor, putative, expressed	Os05g04820	0.68	0.7
Actin, putative, expressed	Os05g36290	−0.97	0
TCP-domain protein, putative, expressed	Os06g12230	0.89	0.29

Gene expression was calculated as log2ratio of shoot vs. root samples. Gene ID was obtained from Michigan State University database.

## Conclusions

A newly-designed rice microarray, the rice OneArray® 22 K microarray, was provided for examining both *japonica* and *indica* subspecies. It was demonstrated this platform displayed high specificity and sensitivity following a comprehensive validation. Based on the unique design, we believe this microarray will be of interest to many researchers in rice studies, especially in important biological and physical controls, and it can be used to facilitate the functional studies toward a hybrid subspecies.

## Methods

### Tissue preparation and total RNA extraction

A three-leaf-stage *japonica* subspecies (Tainung 67, TNG 67) was selected and subjected for total RNA extraction. In general, 100 mg of rice tissue was cut into 5 cm lengths and stored immediately in RNAlater (Invitrogen, Carlsbad, CA, USA) at 4°C until RNA isolation. Rice tissues were homogenized using a RNase-free mortar before performing RNA extraction, and total RNA was isolated from rice roots and shoots using the Qiagen RNeasy Mini kit (Qiagen, Chatsworth, CA, USA) according to manufacture’s protocols.

### cRNA amplification

1 μg of total RNA was converted to double stranded cDNA using reverse transcriptase, and amplified by in vitro transcription using MessageAmpII aRNA Amplification kit (Ambion Inc., Austin, Texas, USA). The synthesized cRNA was subsequently conjugated with Cyanine 5 NHS ester dye (GE Healthcare, Milwaukee, WI, USA). cRNA yield and labeling efficiency was calculated based on ND-1000 spectrophotometer measurements (NanoDrop Technologies, Wilmington, DE, USA). Incorporation rates of 20–60 dye molecules per 1,000 bases (20–33 bases/dye molecule) yielded the most usable data.

### Microarray pre-hybridization

Rice OneArray® microarrays were pre-heated at 60°C for 10 min in hybridization oven. Microarray slides were placed inside a falcon tube containing 100% ethanol, incubated for approximately 15 sec, shaken for 20 sec, and thoroughly rinsed with deionized water to remove any residual ethanol. Next, the microarray slides were fully submerged in an abundant amount of pre-hybridization solution (5X SSPE, 0.1% SDS, and 1% BSA) for 1 hr at 42°C. After 1 hr, slides were transferred to room-temperature distilled water and washed gently for 2 min. Slides were spun dry for 2 min and stored in a dry and dark place until hybridization.

### Microarray hybridization

10 μg of cRNA was fragmented by using RNA Fragmentation Reagent kit (AM#8740, Ambion Inc., Austin, Texas, USA), and then denatured in a PCR machine at 95°C for 5 minutes and held at 60°C. Fragmented cRNA was hybridized on the rice OneArray® (Phalanx Biotech Group, Taiwan) at 50°C for 14–16 hrs. After hybridization, the microarrays were washed sequentially in 2X SSC containing 0.2% SDS solution for 5 min at 42°C, 2X SSC for 5 min at 42°C, and 2X SSC for 5 min at room temperature. Finally, the microarrays were spun dry with a centrifuge for at least one minute and stored dry in the dark until ready for scanning.

### Image scanning

Raw intensity signals for each scanned microarray were captured at 10-μm resolution using GenePix Personal 4000B (Molecular Devices Corporation, Sunnyvale, CA, USA), quantified by GenePix™ Pro 4.0 software (Molecular Devices Corporation, Downingtown, PA, USA), and stored in GPR format. Microarray images were saved as TIFF files. Auto Photomultiplier tube (PMT) settings were selected and adjusted to include the overall feature intensities of Cy5 channel.

### Data processing and statistical analysis

The data from all microarrays was processed using proprietary modeling techniques developed on the Rosetta Resolver® System (Rosetta Biosoftware, Seattle, WA, USA). Raw data is comprised of probe intensities, background values, detected signals, signal-to-noise ratio data, probe identification and gene annotations. After probe filtering based on flag note criteria, normalization of raw intensity was achieved by median scaling and the mean of the technical repeats. The log2 (Ratio) were calculated by pair-wise combination and error weighted average. Significant differentially expressed genes (DE genes) were selected according to its log2 (Ratio) and P-value based on the following criteria; log2 (Ratio) > = 1 and P-value (differentially expressed) <0.05.

### Quantitative real-time RT-PCR

Total RNA was isolated according to the methodology previously described. Primer designs are listed in Additional file 5. 2 ug of total RNA was used to synthesize first-strand cDNA using random hexamers and cDNA reverse transcription kit (Applied Biosystems, Foster City, CA, USA) in a reaction volume of 20 μL. The PCR reactions were performed in a 20 μL volume containing 1× Fast SYBR Green Master Mix, 20 ng cDNA, 10 nM of forward primers, 10 nM of reverse primers using an ABI Prism 7900 HT sequence detection system (Applied Biosystems, Foster City, CA, USA). The amplification conditions were as follows; 95°C for 20 sec, followed by 40 cycles of 95°C for 5 sec and 60°C for 30 sec, and final soak at 4°C. Actin RNA served as the internal control. The levels of 66 mRNA expressions in each of the rice tissue samples were measured by using the 2^-△△Ct^ method. All measurements were performed in triplicate and the experiments were repeated at least twice.

## Competing interests

The authors declare that they have no competing interests.

## Author’s contributions

SL drafted the manuscript. JSC, SCL, CYC, YTH, PHP, and LC collected the genes information and designed those probes. JSC and PHP tested the microarray quality and performed microarray experiment for rice tissues. SCL and LC performed bioinformatic analysis of microarray QC testing, the statistical analysis of expression data and GO functional analysis. JSC and SCL designed and coordinated the study. All authors read and approved the final manuscript.

## Supplementary Material

Additional file 1 **Analyses of up-regulated gene expression between rice shoot and root tissues.** Excel file containing the raw data of up-regulated genes represented for shoot vs. root. Click here for file

Additional file 2 **Shoot-specific expression genes.** Excel file containing the raw data of shoot-specific expression genes represented for comparison between shoot and root. Click here for file

Additional file 3 **Analyses of down-regulated gene expression between rice shoot and root tissues.** Excel file containing the raw data of down-regulated genes represented for shoot vs. root. Click here for file

Additional file 4 **Root-specific expression genes.** Excel file containing the raw data of root-specific expression genes represented for comparison between shoot and root. Click here for file

Additional file 5 **Primer design list for qRT-PCR validation.** Word table containing the selected gene description and primer sequences indicated the primers used for qRT-PCR examination. Click here for file
