# Supplementary material for: Development of a microarray for two rice subspecies: characterization and validation of gene expression in rice tissues
Source: BMC Res Notes. 2014 Jan 8;7:15. doi: 10.1186/1756-0500-7-15 (PMC3891988; doi:10.1186/1756-0500-7-15)
Supplement: Additional file 5 — Primer design list for qRT-PCR validation. Word table containing the selected gene description and primer sequences indicated the primers used for qRT-PCR examination. [file 1756-0500-7-15-S5.docx]

| MSU ID | qPCR primer sequence | Description |
| --- | --- | --- |
| Os01g04120-F | TTACCTACTAGCTGCCTACGTCCG | ZOS1-03 - C2H2 zinc finger protein, expressed |
| Os01g04120-R | ACCTAGCCAGGGAGAGGGATCTTA |  |
| Os01g04800-F | TGGGGCAAGAGGTCTTCCTACTGA | B3 DNA binding domain containing protein, expressed |
| Os01g04800-R | TAGGGCAGGAGTACGCTGATGAAC |  |
| Os01g11550-F | TCCACTTCCATCACGGAATTGTCG | TCP family transcription factor, putative, expressed |
| Os01g11550-R | TGTGCTACCTCCAAACTTTGCTCC |  |
| Os01g48130-F | TTGCTGACCTGCTGTTGCAT | no apical meristem protein, putative, expressed |
| Os01g48130-R | TGGAAGAAGTGCCGGATCA |  |
| Os01g55150-F | TTTCTGGATCAGCAGCGGACGA | bZIP transcription factor domain containing protein, expressed |
| Os01g55150-R | TTCCTCAACGCCTCCTGATGAGCA |  |
| Os01g59660-F | TTCACTGAATCCACCCCTCCTGT | MYB family transcription factor, putative, expressed |
| Os01g59660-R | TCCAACCTGGGGAAACTTTGGAGA |  |
| Os01g66420-F | AGAAGCAGGCGAAAGAAAAGAC | PHD finger protein, putative, expressed |
| Os01g66420-R | TGCACTTTCGAACTAGGCTTGT |  |
| Os01g70310-F | TTGCCCAACCTCATTTCCAA | inducer of CBF expression 2, putative, expressed |
| Os01g70310-R | CCT CATCCTAACTTCGACCGTG |  |
| Os01g72330-F | TCGTCCTACCAAGTGACCACTGTG | OsRR4 type-A response regulator, expressed |
| Os01g72330-R | TCCCTCAATCCAAGCAGCTCCAAG |  |
| Os01g74410-F | GCAAAGGTGTCAGGTTTGCAGC | MYB family transcription factor, putative, expressed |
| Os01g74410-R | TTCAGCCCTGGATGCAGGTAGTTC |  |
| Os02g02290-F | TCATAGCTGACATGCAGCAGATGC | SNF2 family N-terminal domain containing protein, expressed |
| Os02g02290-R | TCGGACCTCATGCCTATAGCTGAA |  |
| Os02g05510-F | ATGTTCAGAACGGAATCGTCGAGG | GATA transcription factor 25, putative, expressed |
| Os02g05510-R | GCCATTTGCAGCAGAAACTGCCT |  |
| Os02g08440-F | AGCCTGGTGGTGAAAGATGGGTA | OsWRKY71 - Superfamily of TFs having WRKY and zinc finger domains, expressed |
| Os02g08440-R | CATCTGAAGTAGGCTCTTGGGCAG |  |
| Os02g29550-F | ATAACAGAACGGTCAAGCCGATT | AP2 domain containing protein, expressed |
| Os02g29550-R | CCTCTGTGATGTCAGGAGCTAACC |  |
| Os02g35600-F | GTCCAACCCTAAACCATCGAAGCA | PHD finger protein, putative, expressed |
| Os02g35600-R | GGCCGCTGTCTTCCTCATCTTTT |  |
| Os02g42380-F | TTCTTCTCTCTACCCCGTGGATCG | TCP family transcription factor, putative, expressed |
| Os02g42380-R | AGCAATTGATGAACCGCACAGGA |  |
| Os02g43790-F | ACCGAAGCGGAGGAAGAGAG | ethylene-responsive transcription factor, putative, expressed |
| Os02g43790-R | TGGTGGAACCAGTGCCATG |  |
| Os02g47810-F | GCCTTGGACTATTTGACTGGAAGC | dof zinc finger domain containing protein, putative, expressed |
| Os02g47810-R | TCATGCCAGCACCCATGAACTG |  |
| Os02g50480-F | TGCTAAGTCCCAGTTTCTGGCAAC | histidine kinase, putative, expressed |
| Os02g50480-R | TGGACGACAAGGACGCCATTCATA |  |
| Os02g51280-F | TGGGGATGAACCTGACCAAAACAC | TCP-domain protein, putative, expressed |
| Os02g51280-R | GGGTGAGGCGAATGAAGCTTGTTG |  |
| Os02g52340-F | TCCGGCTTAGACAAATGAGAGGT | OsMADS22 - MADS-box family gene with MIKCc type-box, expressed |
| Os02g52340-R | AGCATCACCCTGTGCAGACC |  |
| Os02g54830-F | TACCCCCTCCTTTTGCTTGCTAGG | RING-H2 finger protein, putative, expressed |
| Os02g54830-R | TGCTGTTGGATGCAATCGCTAGTT |  |
| Os02g55320-F | GCTGCCAGTATCATGTGACGAC | two-component response regulator, putative, expressed |
| Os02g55320-R | ATGTCCCTGTTTTCCCGCA |  |
| Os03g08960-F | AAACAAGGAGAGGACCTGGCGT | homeobox associated leucine zipper, putative, expressed |
| Os03g08960-R | TCTCGGCTTCATCTTTCTGCTCCA |  |
| Os03g09170-F | CGACAGTGGATTGGTGTGGC | ethylene-responsive transcription factor, putative, expressed |
| Os03g09170-R | CGCCCATTAGCTCCTTGCTATC |  |
| Os03g17570-F | GGTCTGTTACCGAGAAGCTGCATT | response regulator receiver domain containing protein, expressed |
| Os03g17570-R | GCGCACCTTTTTTCCAAAGTTCCT |  |
| Os03g20550-F | GCTCAAGGAAAGAGAAGCGCATGA | OsWRKY55 - Superfamily of TFs having WRKY and zinc finger domains, expressed |
| Os03g20550-R | TTCTCGCCGTACTTCCTCCACT |  |
| Os03g43800-F | TTCAGGCAATGTGCAGTGTGAGT | DIRP family protein, putative, expressed |
| Os03g43800-R | TATCGAGTGCCTCCCCTACTTTGG |  |
| Os03g55270-F | GTTTGGATGAACCCCGCAA | TIP41, putative, expressed |
| Os03g55270-R | GGCAACAAGGTCAATCCGATC |  |
| Os03g60080-F | CATCCCCAGGAACAACAGCA | NAC domain-containing protein 67, putative, expressed |
| Os03g60080-R | GTACATGCCCTGGATATCGTCG |  |
| Os03g60560-F | TTCGCCTAGTTACCGCTGGTCT | ZOS3-21 - C2H2 zinc finger protein, expressed |
| Os03g60560-R | TGACACACACTGCGTCAGTTCAAA |  |
| Os03g61640-F | GGAGAAGGAAGAAGCGCGGAAA | ZOS3-23 - C2H2 zinc finger protein, expressed |
| Os03g61640-R | AGACGGGACTCAAGGTCAACTTCA |  |
| Os04g49450-F | TTCCTAAGGCGCGGAAGCCATACA | MYB family transcription factor, putative, expressed |
| Os04g49450-R | CGTCCTCGGTCCATTTCTCCCTTT |  |
| Os04g57610-F | TGAACCCACAGGAGCAGAACGATG | auxin response factor, putative, expressed |
| Os04g57610-R | GGCTGCTTGCTCATTATCCCCAT |  |
| Os04g58020-F | CGAGCACAGGCTGTTCCTTCTT | MYB_Al protein, putative, expressed |
| Os04g58020-R | AGATACTCCTCCAGTCGCCTTTGC |  |
| Os05g04820-F | TTGGCAACACCCCGTTCTACTG | MYB family transcription factor, putative, expressed |
| Os05g04820-R | TGCTCTGTGGCTCGAATCCCATAC |  |
| Os05g36290-F | ATCCTTGTATGCTAGCGGTCGA | actin, putative, expressed |
| Os05g36290-R | ATCCAACCGGAGGATAGCATG |  |
| Os05g37170-F | TGCCAAGACTGAAAGGCGAT | transcription factor, putative, expressed |
| Os05g37170-R | GCCTTATGCGGCTAGTCTCAAG |  |
| Os05g46370-F | TTCCTTGACTCGGTGGATGTGTTG | bHelix-loop-helix transcription factor, putative, expressed |
| Os05g46370-R | ACGTTCGGGAACGCGAACCTAT |  |
| Os06g03580-F | AGGCTGCCACACAGGATGAT | zinc RING finger protein, putative, expressed |
| Os06g03580-R | GCCTGCCTTTGCTCGTATGT |  |
| Os06g12230-F | TTAGCGGGAAGCCCCACAAGAA | TCP-domain protein, putative, expressed |
| Os06g12230-R | CACACACCCACACAACCACAAAGA |  |
| Os06g24070-F | CAGCTGCGAGATTTCCTGCT | myb-like DNA-binding domain containing protein, expressed |
| Os06g24070-R | TGGCCTATACATTGGACACGG |  |
| Os06g33810-F | GGCCATGAGGTCGTTGAACTATG | zinc finger protein, putative, expressed |
| Os06g33810-R | TCTGGATCTTTCTCAGGGCAAAGT |  |
| Os06g41390-F | CAACAAAGGCTCCTGCACGT | N-terminal asparagine amidohydrolase, putative, expressed |
| Os06g41390-R | GCGCAGCTCACTTTCACCAT |  |
| Os07g29600-F | ACTTTGTCCTCTTACCCCGCCA | zinc finger, C3HC4 type, domain containing protein, expressed |
| Os07g29600-R | CAAGAAAACAACCACTGTGGGTGC |  |
| Os07g38030-F | TGCAGTGGTTTTGCCACGGAGAAG | GRAS family transcription factor domain containing protein, expressed |
| Os07g38030-R | TGGAGCATCACGTCAGCCTAAACA |  |
| Os07g48180-F | TGCGTGATGCTCTCAACGAT | bZIP transcription factor domain containing protein, expressed |
| Os07g48180-R | GGACCTGCTGCATTCCTGTATT |  |
| Os07g48410-F | AGTGTGGAGCATAATCTGCCG | RNA-binding zinc finger protein, putative, expressed |
| Os07g48410-R | AAGGATCGCGAGCAAATCC |  |
| Os07g49460-F | ATAACGGCAGTGGCACTCAAGC | response regulator receiver domain containing protein, expressed |
| Os07g49460-R | GCCTGTGGACTGTCAATCTCAACA |  |
| Os08g03310-F | TTGCAGCATTGAGGACGCCAGT | zinc finger family protein, putative, expressed |
| Os08g03310-R | TCAGGGGATGCAAGGGATGGATT |  |
| Os08g15840-F | TCAAAATTGCCAGGGAATGTGGGT | ankyrin repeat-rich protein, putative, expressed |
| Os08g15840-R | TCCAAACATATGGCGCATGGGTC |  |
| Os08g26880-F | GAACATGGTCACCCAAAGCTGT | bZIP transcription factor domain containing protein, expressed |
| Os08g26880-R | TCTCACGGAGCGCTGATAGC |  |
| Os08g33660-F | ACCAAAGCGCACTGACAATGA | MYB family transcription factor, putative, expressed |
| Os08g33660-R | GCCAACCTCTTTTTCAGGTGG |  |
| Os08g40900-F | TCTTAAATAGAAATTCCCGGTCTGC | auxin response factor, putative, expressed |
| Os08g40900-R | CCACGCTTGTGTACCTTGGTG |  |
| Os09g21180-F | AGAGCCTCCGATCTCAGGTGA | homeobox associated leucine zipper, putative, expressed |
| Os09g21180-R | ACGACTTGGCGCTTTCAGG |  |
| Os09g26400-F | CAGGAGCTACCGTGCAAGCA | zinc finger, C3HC4 type domain containing protein, expressed |
| Os09g26400-R | GGAGTTGTTCTCGTCCAGCCAT |  |
| Os09g29960-F | CGACACCGATCCATCACTGTTCCT | dof zinc finger domain containing protein, putative, expressed |
| Os09g29960-R | TGCTAACCATCCTCTCGTTCGTCC |  |
| Os09g30400-F | GCGAAATTGCGTCTTTTGTGGGT | OsWRKY80 - Superfamily of TFs having WRKY and zinc finger domains, expressed |
| Os09g30400-R | ATTCCCCACCACAGCAAACCAG |  |
| Os09g32510-F | TGCAACAAGGTGGTTGGCAAGG | BHLH transcription factor, putative, expressed |
| Os09g32510-R | TCGACCTGCTGCTGCAACGATT |  |
| Os09g33670-F | GGGGAAAGCGAATCTGAGCCAAAT | zinc finger, C3HC4 type domain containing protein, expressed |
| Os09g33670-R | TGGCAGTAGAACTAGAAGGCGCAT |  |
| Os09g35880-F | TGATCTTAATATGAGACCCGTCCG | B-box zinc finger family protein, putative, expressed |
| Os09g35880-R | ATCCACGCCCTGAGTCTGTG |  |
| Os09g36250-F | TTGCTTTGATGCACGCCTAGCTAA | MYB family transcription factor, putative, expressed |
| Os09g36250-R | TGGAGCCGCTGCTGTTTCGAAT |  |
| Os10g33940-F | TTGGTCGCACCATTGATTTGT | auxin response factor 18, putative, expressed |
| Os10g33940-R | CAGCCAGCCGACCATACAGT |  |
| Os10g42130-F | GCGTGATCAAGAGGCACGA | no apical meristem protein, putative, expressed |
| Os10g42130-R | TGCTGATCATTCCTTTCGCC |  |
| Os12g13170-F | TCGCAGATCTAGGTTGCGTAAG | transcription factor, putative, expressed |
| Os12g13170-R | AACTTCAGCACGTTGAGCCA |  |
| Os12g31840-F | GCAAAAGGAGGTTTCAAAGGCACC | ZOS12-05 - C2H2 zinc finger protein, expressed |
| Os12g31840-R | TTGAGTCAAACGTTGATCCACACG |  |
